# Supplementary material for: From discouragement to self-empowerment. Insights from an ethnolinguistic vitality survey among the Kashubs in Poland
Source: PLoS One. 2020 Aug 20;15(8):e0237395. doi: 10.1371/journal.pone.0237395 (PMC7446787; doi:10.1371/journal.pone.0237395)
Supplement: Attachment — Submitted filename: Rebuttal letter Olko.pdf [file pone.0237395.s002.pdf]

Kraków, 16.07.2020

Dear Dr. Anastassia Zabrodskaja,
Academic Editor
PLOS ONE

Firstly, we would like to thank the reviewers for all their comments and recommendations. We found all of them to be very helpful for improving our paper. We have implemented all of the recommendations and, additionally, made some further improvements that build on these recommendations.

In full accordance with the recommendations of Reviewer #2 and Reviewer #3, we have broadened and depended the introduction to the Kashubian community in the initial section of the paper, focusing on its socio-political situation and status. We have also added more socio-political framing regarding the current situation in Poland in terms of the language rights of national and ethnic minorities, including the state's attitude toward them and the implications of their current legal status. Contextualizing the findings in this way facilitates a deeper understanding of the results discussed later in the paper and their significance. We have also included more references to the most recent relevant publications, including a book published in 2020 that was unavailable when we prepared the first draft of the paper. At the end of the "Introduction" we have also added a short description of the general structure of the paper, as a sort of "roadmap" for readers.

In the section "Context of the study" we have added to the historical overview of the Kashubs, with a special focus on post-1945 policies toward the group, which are important for understanding their present situation. We have also provided a more concise description of the group's situation and vitality after 1989, as well as the changes that were set in motion after the recognition of Kashubian as a regional language in 2005. This includes an up to date overview of current spaces of language use and development. Moreover, these amendments better contextualize and explain the 2011 census data as it relates to the Kashubs and the Kashubian language. In full accordance with the recommendation of Reviewer #2, we have also provided a description of different types of Kashubian speakers and their language proficiencies, based both on earlier research (with additional references included) and our own knowledge of the topic. The latter embraces the emic knowledge and experience of one of the co-authors, who is a Kashubian scholar, teacher, activist and publicist. We decided to complement this description on kinds of speakers and spaces of language use by deepening the presentation and discussion of the related quantitative data obtained in our survey and described in the section "Results". This improvement offers sociolinguistic insight that is required for better understanding the profile of our survey participants and for interpreting the survey results. Thus, we have described with more precision and in more detail participants' self-reported oral and written skills in Kashubian and Polish, along with their domains of language use, adding an additional Table 1 that illustrates our data.

Finally, as suggested by Reviewer #1, we have strengthened our arguments in the section "Discussion and conclusions" and we have provided more references to other similar or relevant studies. Thus, we have included more insights from additional studies on the Rejection-Identification Model, which is particularly relevant for our case study and our discussion; we have also added a substantial section on the concept of empowerment and

previous approaches to the theme of empowerment of ethnic minorities and, particularly, of speakers of minority/indigenous/endangered languages. In accordance with this discussion and our theoretical framework, we have also decided to change *empowerment* to *self-empowerment* in the title and relevant parts of the paper, as it describes more adequately the hypothesis and the interpretation of findings developed in this study, thus strengthening our arguments. Finally, we have consolidated our arguments with regard to the profile of our survey participants who represent an active and engaged sector of the Kashubian community'.

We have also corrected spelling and grammar issues in the figures accompanying the paper and we provide them in a much better quality in the form of TIFF files. We also changed the type of charts presented in Figures 2, 3 and 4 to stacked bar charts, which makes them easier to read. Regarding other formatting details, including references, we follow the guidelines of the journal. Finally, we have implemented other minor editing improvements throughout the paper.

Summing up, we have fully followed the recommendations of the reviewers, with with which we fully agree. In addition to implementing their direct suggestions, we have also introduced some additional improvements to other parts of the paper that relate to these recommendations and contribute to the overall coherence of the paper, the strength of our arguments and the interpretation of the results.

With kind regards,

Justyna Olko
